# Cappable-seq RNA-sequencing data sets of *Escherichia coli* K-12 MG1655 treated with novobiocin, tetracycline, and rifampicin

**DOI:** 10.1128/mra.01194-24

**Published:** 2024-12-27

**Authors:** Alexander Balkin, Andrey Plotnikov, Tatiana Konnova, Elena Shagimardanova, Hamza Hamo, Yuri Gogolev, Natalia Gogoleva

**Affiliations:** 1 Kazan Institute of Biochemistry and Biophysics, Kazan Scientific Center of Russian Academy of Sciences, Kazan, Russia; 2 Institute for Cellular and Intracellular Symbiosis, Ural Branch of the Russian Academy of Sciences, Orenburg, Russia; 3 Center for Personalized Medicine, Loginov Moscow Clinical Scientific Center, Moscow, Russia; 4 Skolkovo Institute of Science and Technology, Skolkovo, Russia; 5 Institute of Fundamental Medicine and Biology, Kazan Federal University, Kazan, Russia; 6 Research Department for Limnology, Mondsee, Universität Innsbruck, Mondsee, Austria; University of Maryland School of Medicine, Baltimore, Maryland, USA

**Keywords:** Cappable-seq, *Escherichia coli* K-12, transcription start sites, antibiotic resistance

## Abstract

Mapping transcription start sites and determining their activity remain a challenging task even for well-studied organisms. Here, we present Cappable-seq RNA sequencing data of *Escherichia coli* K-12 MG1655 after treatment with three antibiotics with various spectra of action that may expand the range of mapped transcription start sites for this model organism.

## ANNOUNCEMENT

Standard RNA sequencing does not reveal the full complexity of transcriptomes (1–3). The Cappable-seq method was developed to identify transcription start sites (TSSs) (1). Here, we announce Cappable-seq RNA sequencing data expanding the range of mapped *Escherichia coli* K-12 MG1655 TSSs during adaptation to antibiotics.

*E. coli* K-12 cultures were grown in Luria-Bertani broth at 37°C with shaking (190 rpm). All experiments were performed in triplicate. In the middle of the exponential phase (OD_600_ = 0.6), tetracycline, rifampicin, or novobiocin was added to the experimental cultures at four different concentrations. Concentrations that, after 1 hour of treatment, reduced the optical density of the experimental cultures by 0.2 units compared to the control were selected for further experiment. According to the OD/CFU correlation plot, the observed difference corresponded to approximately a 50% reduction in CFU count. The determined concentrations were 12.5, 50, and 100 mg/L for tetracycline, rifampicin, and novobiocin, respectively. Initial changes in the transcriptome were the focus of the experiment. The bacterial cells were fixed 15 min after the treatment with an equal volume of RNA-stabilizing solution (19% ethanol and 1% phenol, pH 5.5) on ice for 30 min (4). The time of fixation coincided with the divergence of the growth curves of the control and experimental cultures. Bacterial cells were harvested by centrifugation and used for RNA extraction using TRIzol reagent and TURBO DNA-free kit according to the manufacturer’s instructions (Thermo Fisher Scientific, Waltham, MA, USA).

The total RNA (5 µg) was capped with 3′ desthiobiotin-GTP using the Vaccinia Capping System (New England Biolabs, Ipswich, MA, USA) (1). The Capped RNA was purified by Zymo Research Clean and Concentrator-5 column (Zymo Research, Irvine, CA, USA) and fragmented by heating at 94°C for 5 min in polynucleotide kinase buffer. The 3′-phosphates were removed from the RNA with T4 polynucleotide kinase at 37°C for 15 min. The capped RNA was captured with hydrophilic streptavidin magnetic beads (NEB), eluted with biotin, and purified using AMPure XP beads (Beckman Coulter, Brea, CA, USA).

Libraries were generated using the NEBNext small RNA library prep kit (NEB), assessed using a high-sensitivity Bioanalyzer DNA chip (Agilent, Santa Clara, CA, USA), and quantified by qPCR. Sequencing (75 bp paired end) was performed on the HiSeq 2500 instrument (Illumina, San Diego, CA, USA).

Demultiplexed single-end reads were trimmed for adapters using bbduk version 38.76 (http://sourceforge.net/projects/bbmap/) with “ktrim = r,” “k = 23,” “mink = 11,” “hdist = 1,” “tpe,” “tbo,” and “minlen = 25” parameters. In further work, default parameters were used for all software. SortMeRNA version 4.3.6 (5) was used to remove ribosomal RNA sequences. Clean reads were mapped against the *E. coli* K-12 MG1655 genome (GCF_000005845.2) with Bowtie2 version 2.3.5.1 (6) in local mode (L-16). The files were converted to bam format using SAMtools version 1.6 (7).

A total of 269,956,414 reads were obtained. Table 1 shows the results of mapping clean reads onto the *E. coli* K-12 MG1655 genome after the removal of ribosomal sequences. The announced data provide a resource for mapping new transcription start sites and studying transcriptome changes in *E. coli* in the presence of certain antibiotics.

**TABLE 1:** General summary of sequencing data

Sample	Total reads	Clean reads after the removal of rRNA sequences	Mapped clean reads	SRA accession no.	GEO accession no.
K1	17.890.909	16.737.192	15.552.554 (92.92%)	SRX17858408	GSM6631703
K2	29.283.453	27.754.747	25.888.823 (93.28%)	SRX17858411	GSM6631706
K3	20.548.314	19.390.926	17.663.812 (91.09%)	SRX17858408	GSM6631709
Nb1	19.256.272	17.485.438	15.936.782 (91.14%)	SRX17858417	GSM6631712
Nb2	21.166.221	18.625.116	17.243.257 (92.58%)	SRX17858420	GSM6631715
Nb3	22.887.762	16.402.791	12.864.661 (78.43%)	SRX17858423	GSM6631718
Rif1	20.960.312	18.878.950	13.486.768 (71.44%)	SRX17858426	GSM6631721
Rif2	19.501.020	17.716.769	12.875.042 (72.67%)	SRX17858429	GSM6631724
Rif3	21.534.377	17.417.098	10.498.505 (60.28%)	SRX17858432	GSM6631727
Tet1	26.101.901	24.391.119	22.853.131 (93.69%)	SRX17858435	GSM6631730
Tet2	25.534.068	24301904	22.402.229 (92.18%)	SRX17858438	GSM6631733
Tet3	25.291805	22.179.933	19.756.234 (89.07%)	SRX17858441	GSM6631736

## Data Availability

Cappable-seq RNA sequencing data have been deposited in the NCBI SRA database and were available through BioProject accession number PRJNA889731. The results of mapping the processed reads have been deposited in GEO under data set accession number GSE215300.
